# Spinal intradural metastasis from scapular Ewing sarcoma

**DOI:** 10.1186/s13104-015-1263-0

**Published:** 2015-07-08

**Authors:** Dissanayake Mudiyanselage Priyantha Udaya Kumara Ralapanawa, Kushalee Poornima Jayawickreme, Ekanayake Mudiyanselage Madhushanka Ekanayake, Kulatunga Wijekoon Mudiyanselage Pramitha Prabhashini Kumarihamy

**Affiliations:** Department of Medicine, University of Peradeniya, Peradeniya, Sri Lanka; University Medical Unit, Teaching Hospital Peradeniya, Peradeniya, Sri Lanka

**Keywords:** Ewing sarcoma, Flaccid paralysis, Scapular bone neoplasm, Spinal metastasis, Sri Lanka

## Abstract

**Background:**

Ewing sarcoma is a primary bone neoplasm, which is a high grade aggressive small round blue cell tumour, and is currently recognized as a part of the Ewing family of tumours. It is the most lethal bone tumor, and is a rare malignant bone tumor accounting for 10% of all primary bone tumors, and 6% of malignant bone tumors. It has an average annual incidence of 3 per 1 million, found almost exclusively in Caucasians. It commonly occurs in long bones and pelvis but rarely involves the scapula. 85% of cases have metastasis within 2 years of diagnosis, rarely involving the meninges.

**Case presentation:**

We report a case of a 25 year old Sinhalese Sri Lankan female, presenting with a 1 day history of bilateral lower limb weakness and urinary incontinence. She had a sensory level with flaccid paralysis of lower limbs and a painless bony lump in the left dorsal scapula. Investigations showed scapular primary Ewing sarcoma giving rise to spinal intradural metastasis. For the best of our knowledge this is the first reported case of a scapular Ewing sarcoma with spinal intradural metastasis presenting with lower limb paralysis.

**Conclusion:**

Intradural spinal metastasis of Ewing sarcoma presenting with lower limb weakness, without a history of pain, though rarely, can be the first presentation, and can rapidly progress to brainstem involvement and death.

## Background

Ewing sarcoma (ES) is a primary bone neoplasm which is a high grade aggressive small round blue cell tumour, and is currently recognized as a part of the Ewing family of tumours. It was first described by famed pathologist James Ewing in 1921 [1, 2]. It commonly demonstrates reproducible staining of CD99 and translocations of the EWS gene. This neoplasm is usually associated with near certain metastasis at the time of diagnosis with a high rate of mortality. Occurrence of ES is frequently seen in the second decade of life in the diaphysis of long bones and pelvis; and has an average annual incidence of about 3 per 1 million. It has a slight predominance in males which accounts for 61% of the cases diagnosed, and is found almost exclusively in Caucasians who represented 92% of the cases [2].

## Case presentation

A 25 year old Sinhalese Sri Lankan female presented with a 1 day history of bilateral lower limb weakness, and numbness with urinary incontinence. She had no back pain and no history of constitutional symptoms such as fever, loss of appetite, or recent subjective weight loss.

On examination she had atonic lower limbs, with absent muscle power, and absent bilateral lower limb reflexes below knee level, with sensory impairment up to T6 level. She had no spinal deformities or tenderness, and no papilloedema. Upper limb examination was unremarkable except for a hard non tender bony mass on the left scapular region. She had a blood pressure of 140/80 mmHg, pulse rate of 78 beats per minute and had no respiratory compromise.

**Figure 1:**
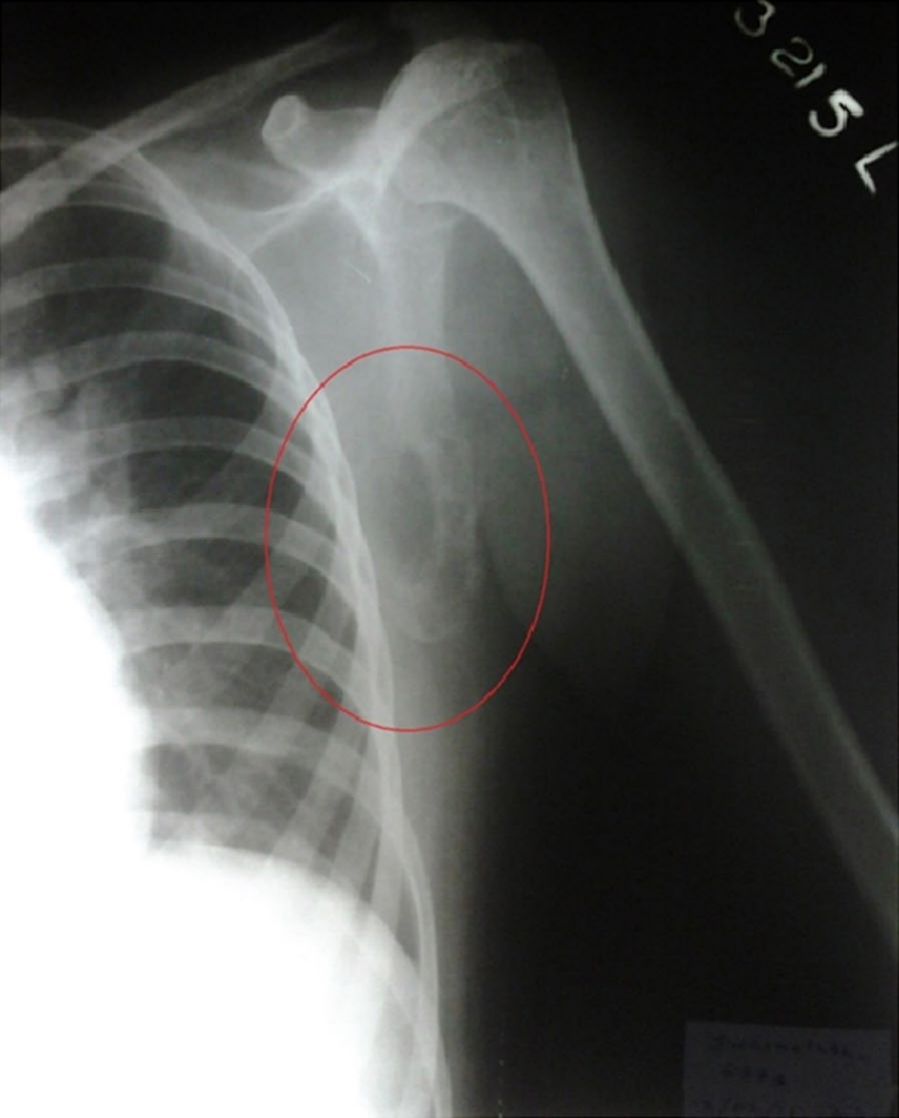
Radiograph of the *left* scapula. Radiograph of the *left* scapula showing a bony mass with multiple lytic lesions in the lateral and dorsal aspects.

**Figure 2:**
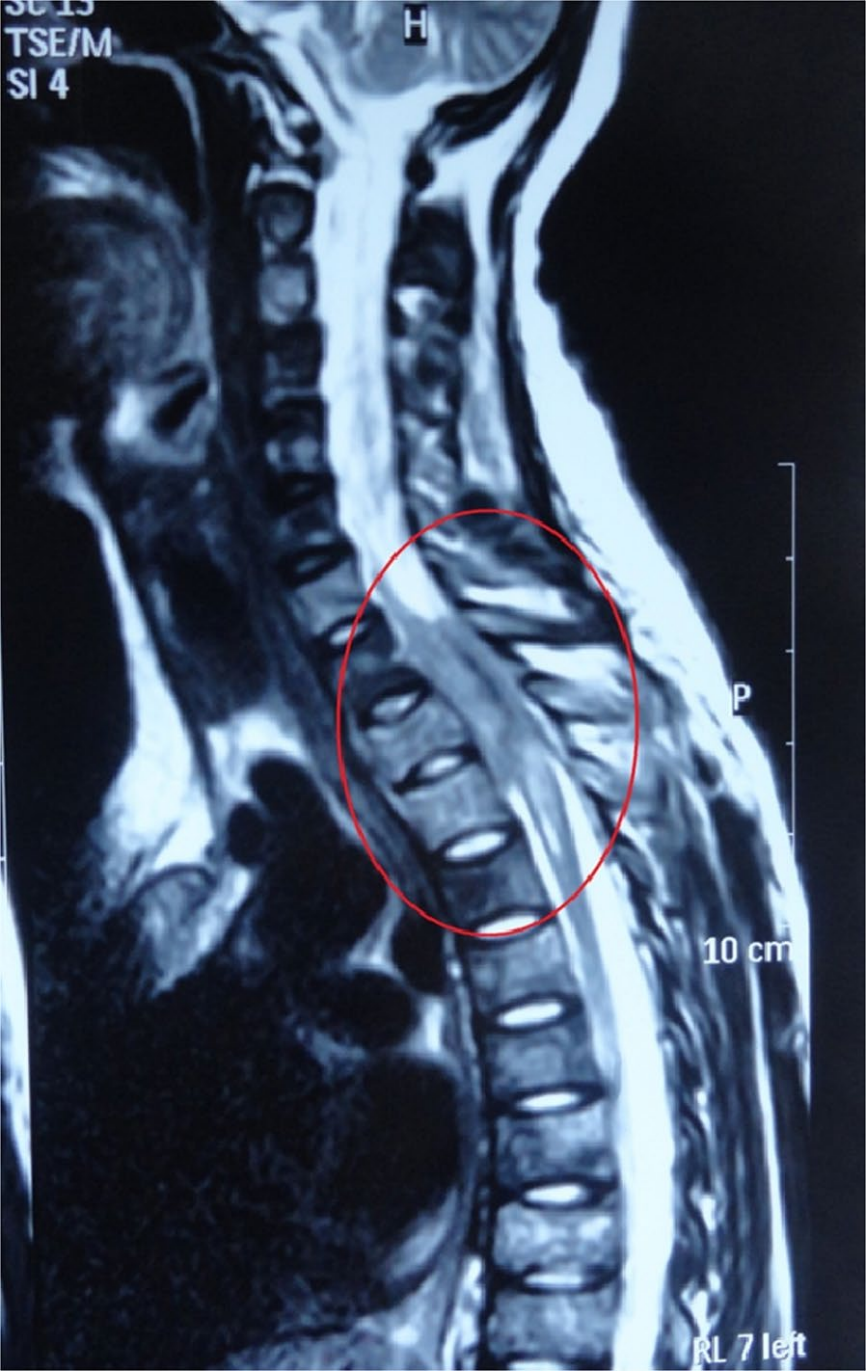
MRI image of cervical and thoracic spine. There is an intradural extra-medullary mass with an extraspinal component at C7-D2 level causing cord compression and there is a paraspinal component at this level which shows some degree of enhancement. Intervening intervertebral discs are normal.

She was investigated with a suspicion of metastatic disease and X-ray of the left shoulder showed a soft tissue and bony mass on the dorsal aspect of the left scapula with multiple lytic lesions suggestive of a primary bone neoplasm (Figure 1), but chest radiograph, ultrasound scan of the neck, and Computed tomography (CT) of abdomen were normal. Magnetic resonance imaging (MRI) of the spine showed an intradural extramedullary mass with an extra spinal component at C7-T2 level causing severe cord compression (Figure 2), hence intravenous dexamethasone regimen was started.

Ultrasound guided core needle biopsy from the left scapular mass showed malignant small round blue cell tumour suggestive of Ewing sarcoma. Blood investigations showed Heamoglobin of 9.2 g/dl, white blood cell count of 12 × 10^3^/μl, Erythrocyte sedimentation rate (ESR)of 150 mm/h, C-reactive protein level of 96 mg/l, normal liver enzyme levels and liver functions tests, and serum alkaline phosphate level of 173 μ/l. Her blood picture showed increased rouleaux formation with anaemia of chronic disease.

She had no improvement of symptoms following treatment with dexamethasone. Before implementing on oncological management, 3 days after onset of symptoms, she developed sudden onset progressive ascending neurological impairment with upper limb and bulbar involvement, and unfortunately resulted with respiratory failure and death.

## Discussion

ES, which is the most lethal bone tumor, is a rare malignant bone tumor accounting for 10% of all primary bone tumors, and 6% of malignant bone tumors [3]. The commonest symptom of ES is a dull to severe pain which becomes persistent from about 1 month prior to diagnosis, and is seen in approximately 70% cases, unlike in this patient who had a painless mass [3]. Other features are fever, anemia, leukocytosis and increased ESR. ES is thought to be neuroectodermal in origin. More than 80% of ES occur during the first two decades of life, wherein Krieun has described the distribution of ES varying with age mirroring the distribution of marrow [4].

ES more commonly occurs in tubular bone than in flat bone, and the commonest affected site is the pelvis (25%), followed in order of frequency by ribs, femurs, spine, tibias, fibulas, scapulas and other bones [4]. The primary site of tumor in this case was the scapula. However, malignant tumor of the scapula is rare, which may comprise of chondro-sarcoma, synovial sarcoma, ES, and metastasis [5]. This case presented with spinal intradural metastasis, whereas primary malignant sarcoma of the spine is rare accounting for 3.5–14.9% of primary bone sarcomas [6]. Metastasis occurs in up to 85% of patients within 2 years of diagnosis. Metastasis occurs to lungs (85%), bones (69%), pleura (46%), lymph nodes (46%), dura and/or meninges (27%) and central nervous system (12%) respectively [7].

Central nervous system involvement which is seen in 10–35% of patients occurs by either spinal cord compression or meningeal dissemination [8]. Most previous reports of central nervous system metastasis of ES showed involvement of the bony calvarium or brain paranchyma. Most such lesions were found to be located in an intracranial or extradural site, making epidural and intradural metastasis even rarer [9]. The commonest malignancies involving the spinal epidural space are metastatic lymphomas, nerve sheath tumors, meningiomas, hemangiomas and metastasis from systemic malignancies. Primary extra-skeletal ES occurring in a paravertebral location with a predilection to infiltrate through neural exit foramina have been rarely reported [10].

Microscopic characteristics typical of ES include, Periodic acid-Schiff positive, diastase-digestible cytoplasmic material, clear cytoplasm with indistinct cellular borders, and the uniformity of small oval blue nuclei [11]. A histology of small round cell bone tumor can be further confirmed as ES by immunohistochemical and cytogenetic analysis, which shows consistent chromosomal anomaly, the reciprocal translocation *t*(11; 22)(q24; q12) [12]. ES is classically described radiologically as a central, diaphyseal, lytic or rarely sclerotic tumor, with “onion skin” type periosteal reaction affecting a long bone associated with soft tissue mass [13]. MRI, which is superior to CT, is frequently associated with a prominent soft tissue mass that contains areas of necrosis or hemorrhage [3].

The successful treatment of patients with ES requires systemic chemotherapy in conjunction with either surgery or radiation therapy or both modalities for local tumor control. Prognosis depends on the size and location of the tumor, presence or absence of tumor metastasis, tumor response to therapy, age, and disease relapse, out of which the presence of distant metastasis having the worst prognosis as seen in this patient. The current long term survival of ES is found to be 60–70%. However, patients with metastasis only have a long term survival of 20%, despite aggressive treatment. Histological grades have no prognostic significance, but the presence of fever, anaemia, leukocytosis, elevated ESR, and lactate dehydrogenase have been reported to indicate more extensive disease and a worse prognosis [13].

## Conclusion

Intradural spinal metastasis of ES presenting with lower limb weakness, without a history of pain, though rarely, can be the first presentation, and can rapidly progress to brainstem involvement and death.

### Consent

Written informed consent was obtained from the deceased patient’s mother for publication of this case report and accompanying images. A copy of the written consent is available for review by the Editor-in-chief of this journal.

## Abbreviations

ES: Ewing sarcoma
CT: computed tomography
MRI: magnetic resonance imaging
ESR: erythrocyte sedimentation rate
